# Implementation of the Diabetes Prevention Program in Georgia Cooperative Extension According to RE-AIM and the Consolidated Framework for Implementation Research

**DOI:** 10.1007/s11121-023-01518-0

**Published:** 2023-03-17

**Authors:** Hannah K. Wilson, Caroline Wieler, Darci L. Bell, Ajit P. Bhattarai, Isaura M. Castillo-Hernandez, Ewan R. Williams, Ellen M. Evans, Alison C. Berg

**Affiliations:** 1 Department of Nutrition, Dietetics and Exercise Science, Concordia College, Moorhead, MN 56562 USA; 2 Department of Nutritional Sciences, University of Georgia, Athens, GA 30602 USA; 3 Department of Organizational Learning and Performance, Idaho State University, Pocatello, ID 83209 USA; 4 Human Movement Sciences Research Center, School of Physical Education and Sports, University of Costa Rica, San José, 11502 Costa Rica; 5 Department of Health Sciences and Research, Medical University of South Carolina, Charleston, SC 29425 USA; 6 Department of Kinesiology, University of Georgia, Athens, GA 30602 USA

**Keywords:** Type 2 diabetes, Prediabetes, Preventive health programs, Community health, Community health education, Implementation science, Qualitative research

## Abstract

Increased dissemination of the CDC’s Diabetes Prevention Program (DPP) is imperative to reduce type 2 diabetes. Due to its nationwide reach and mission to improve health, Cooperative Extension (Extension) is poised to be a sustainable DPP delivery system. However, research evaluating DPP implementation in Extension remains scant. Extension professionals delivered the DPP in a single-arm hybrid type II effectiveness-implementation study. Semi-structured interviews with Extension professionals were conducted at three time points. The Consolidated Framework for Implementation Research (CFIR) guided interview coding and analysis. Constructs were rated for magnitude and valence and evaluated as facilitators or barriers of RE-AIM (Reach, Effectiveness, Adoption, Implementation, Maintenance) outcomes. The program reached 119 participants, was adopted by 92% (*n* = 12/13) of trained Extension professionals and was implemented according to CDC standards: all programs exceeded the minimum 22-session requirement (26 ± 2 sessions). The program was effective in achieving weight loss (5.0 ± 5.2%) and physical activity (179 ± 122 min/week) goals. At post-intervention, eight professionals (67%) had begun or planned to maintain the intervention within the next 6 months. Several facilitators were identified, including Extension leadership structure, organizational compatibility, and technical assistance calls. Limited time to recruit participants was the primary barrier. Positive RE-AIM outcomes, facilitated by contextual factors, indicate Extension is an effective and sustainable DPP delivery system. Extension and other DPP implementers should plan strategies that promote communication, the program’s evidence-base, recruitment time, and resource access. Researchers should explore DPP implementation in real-world settings to determine overall and setting-specific best practices, promote intervention uptake, and reduce diabetes.

**Supplementary Information:**

The online version contains supplementary material available at 10.1007/s11121-023-01518-0.

## Introduction

With 96 million American adults living with prediabetes and 5–10% of these individuals developing type 2 diabetes mellitus (T2DM) each year, increased dissemination of evidence-based T2DM prevention interventions is imperative (Glechner et al., 2018). The Centers for Disease Control and Prevention’s (CDC) National Diabetes Prevention Program (DPP) aims to increase screening for and detection of prediabetes and T2DM and to increase dissemination of and access to the diabetes prevention. The DPP is a 12-month lifestyle change intervention designed to reduce T2DM risk through diet, exercise, and lifestyle changes (Knowler et al., 2002, 2009). DPP clinical trials resulted in reduced rates of T2DM up to 58% in individuals with prediabetes (Allaire et al., 2020; Knowler et al., 2002, 2009). Since the initial DPP clinical trial, nearly 20 years of translational research has demonstrated similar results can be achieved using trained lay leaders in a variety of settings if critical components are upheld (i.e., use of approved curriculum, program duration, frequency of sessions) (Centers for Disease Control and Prevention, 2018; Ali et al., 2012).

Cooperative Extension (here forth, “Extension”), with its over 100-year history of providing health education interventions, presence in almost every county in every state, and trained personnel (i.e., state-level Extension leaders with health program implementation expertise: Extension Specialists), is poised as an effective platform for DPP dissemination and implementation (Franz & Townson, 2008; Molgaard, 1997). In the state of Georgia, there are 159 counties, with 57 having a county-based Extension professional that specializes in health and wellness (University of Georgia, 2023). At present, 31 Extension organizations, representing 17 U.S. states, are CDC-recognized DPP providers (CDC, 2023). While this number is growing, this is far fewer than the potential 50 states and additional U.S. territories that could be DPP providers.

While CDC is tracking overall effectiveness of the DPP among CDC-recognized providers (Ely et al., 2017), little is known about context-specific effectiveness and implementation and which organization types are uniquely positioned to succeed in effective and sustainable DPP delivery. Damschroder et al. (2017a) published a rigorous evaluation of DPP implementation in the Veterans Health Administration (VA) system and revealed that the DPP’s strong evidence base and committed leadership facilitated VA implementation with time as a primary implementation barrier. Beyond this important work in the VA system, a paucity of data exists exploring implementation of the DPP adhering to CDC standards in various community contexts, especially Extension (Nicole et al., 2021; CDC, 2018). Additional implementation research targeting translation of the DPP to Extension and other delivery systems is imperative to help reduce T2DM (Whittemore, 2011). The authors conducted a 12-month hybrid type 2 effectiveness-implementation study of the DPP in Georgia Extension from January 2020-March 2021 using a single-arm, multi-site design (Curran et al., 2022; Damschroder et al., 2017a; Swindle et al., 2019).

## Purpose and Objectives

The study purpose was to rigorously evaluate community translation of the DPP by Georgia Extension according to CDC standards using the 2016 Prevent T2 curriculum (CDC, 2018). To encourage comparability across translational studies of evidence-based programs and to meet the call for greater use of the Consolidated Framework for Implementation Research (CFIR) in implementation science, the Reach, Effectiveness, Adoption, Implementation, and Maintenance (RE-AIM) framework and the CFIR were selected to evaluate DPP implementation and effectiveness (Glasgow et al., 2019; King et al., 2019; Kirk et al., 2016; CFIR Research Team-Center for Clinical Management Research, 2019; Swindle et al., 2019; Varsi et al., 2015). Moreover, to encourage comparability across DPP implementation contexts, we modeled our work after Damschroder et al.’s research on the DPP implemented in the VA, the only comparable literature to date (Damschroder et al., 2015; Damschroderet al., 2017a). Thus, our specific objectives were to (1) evaluate implementation of the DPP in Georgia Extension using RE-AIM, and (2) identify implementation barriers and facilitators that influenced RE-AIM outcomes using the CFIR.

## Methods

### DPP Participants

DPP participants were overweight or obese adults (18–75 years) with or at high risk for prediabetes and without any major comorbidities or physical disabilities (n = 88). Program participants were recruited via word of mouth, physician referrals, new or existing community collaborations/relationships, local employers, flyers, newspaper ads, radio announcements, social media advertisements, and in-person informational sessions. All DPP participants were recruited to participate in the research study by Extension professionals at initial program sessions or via email or phone after registering to participate in the DPP. All 13 Extension professionals implementing the DPP in 2020 were recruited to participate in the implementation study during regular technical assistance calls and via email. All methods and procedures were approved by the University’s Institutional Review Board on Human Subjects. All participants provided informed consent. Study components are reported according to the Standards for Reporting Implementation Studies (StaRI) checklist (Pinnock et al., 2017).

### Implementer Training

In October 2019, 13 Georgia Extension professionals with health and wellness specialty attended the CDC-approved Emory University Diabetes Training and Technical Assistance Center two-day DPP lifestyle coach training that is required for individuals implementing the DPP (Emory University, 2021). Researchers led a third training day on Extension context-specific DPP implementation, including data reporting, research measures, and standards required to obtain CDC recognition as a DPP provider (weight loss, PA, attendance, session length and frequency, total program length) (CDC, 2018).

Following the training, technical assistance calls with Extension professionals were held weekly by the Extension Specialist and graduate research assistant, then decreased to every other week in mid-June 2020 per Extension professionals’ recommendation. Technical assistance calls covered program support resources available from the Diabetes Training and Technical Assistance Center (Emory University, 2021) and the research team, updates from the research team, and time to discuss and explore intervention and/or research study successes and challenges (e.g., recruitment, implementation questions, participant barriers).

### DPP Implementation

Twelve Extension professionals began implementing the in-person DPP in January 2020 to March 2020 in thirteen Georgia counties (7 metropolitan and 6 nonmetropolitan counties) (U.S. Economic Research Service, 2020). One Extension professional delivered programs in two counties. In June 2020, one Extension professional resigned and the 13^th^ trained Extension professional not implementing a DPP at the start of the study took over that cohort. Upon the onset of the COVID-19 pandemic in March 2020, all DPPs transitioned from in-person to distance learning formats (12 groups via Zoom Web Conferencing, and one group via FreeConferenceCall.com®) for the remainder of the 12-month program (FreeConferenceCall.com, 2021; Zoom Video Communications, 2021). Conversion to distance learning followed the CDC’s guidance on transition to distance-learning modes in response to COVID-19 (CDC, 2021). Distance delivery support was provided by the research team during technical assistance calls, one-on-one technology support, and additional materials accessible through the university’s learning management system, and from the Diabetes Training and Technical Assistance Center and CDC webinars available to all DPP providers.

### Implementation Evaluation

The implementation evaluation utilized quantitative program participant outcome data, DPP program reports, and qualitative semi-structured interviews with DPP implementers (Extension professionals) to identify implementation barriers and facilitators according to the CFIR and their influence on RE-AIM outcome domains.

The RE-AIM framework was used to evaluate quantitative implementation outcomes and effectiveness (Glasgow et al., 2019). Reach (R) was calculated as the number of eligible individuals who participated (attended at least one session) compared to the number of interested participants (124 that attended informational sessions only). Effectiveness (E) was measured per CDC intervention goals as participant percent weight loss (≥ 5% of initial body weight) and physical activity (150 min per week of moderate activity). Adoption (A) was measured as the number of trained Extension professionals who implemented the program. Implementation (I) was assessed by fidelity to important program components (program and session duration, frequency and number of sessions) according to reports completed by Extension professionals following each session (Table 1). Maintenance (M) was defined as the number of Extension professionals that had already begun another DPP at the end of the present study or had a start date for a new DPP planned within the next 6 months.

**Table 1:** RE-AIM outcomes of the DPP in Extension and select associated implementation barriers and facilitators

**RE-AIM domain**	**Outcomes**	**CFIR and additional implementation constructs**		
		***Facilitator***	***Barrier***	***Mixed***
**Reach**	• 124 individuals screened • 119 eligible for program • 88 eligible for research	• External change agents • Structural characteristics	• Time	
**Effectiveness**	• 46.7% of participants met 5% weight loss goal (M ± SD: 5.2% ± 5.0%) • 56.7% met PA goal (M ± SD: 179 ± 122 min/week)	• Evidence strength & quality • Relative advantage • Compatibility • Organizational incentives & rewards • Goals & feedback • Participant receptivity	• Complexity	
**Adoption**	• 12 out of 13 (92%) of Extension professionals trained immediately adopted	• Intervention source • Evidence strength & quality • Patient needs & resources • Implementation climate • Tension for change • Organizational incentives & rewards • Leadership engagement • Opinion leaders • Formally appointed internal implementation leaders • Champions	• Cost	• Structural Characteristics
**Implementation**	• Avg sessions implemented: 26 (range: 25–27)	• Evidence strength & quality • Relative advantage • Networks & communications • Implementation climate • Tension for change • Organizational incentives & rewards • Goals & feedback • Learning climate • Readiness for implementation • Leadership engagement • Access to knowledge & information • Individual identification with organization • Other personal attributes • Opinion leaders • Formally appointed internal implementation leaders • Champions • External change agents • Implementation strategy • Agent networks	• Complexity • Cost • Time • COVID	• Structural Characteristics
**Maintenance**	• 5 Extension professionals started 6 new DPP cohorts (virtual) • 1 Extension professional had started 1 new in-person DPP cohort • 2 Extension professionals planned for 2 new in-person cohort; these were implemented as planned after the conclusion of the study	• Evidence strength & quality • Relative advantage • Implementation climate • Compatibility • Organizational incentives & rewards • Learning climate • Leadership engagement • Access to knowledge & information • Individual stage of change • Formally appointed internal implementation leaders • Implementation strategy • Agent networks		

*RE-AIM* Reach, Effectiveness, Adoption, Implementation, and Maintenance, *CFIR* Consolidated Framework for Implementation Research

The CFIR guided qualitative evaluation of the implementation process. A trained graduate student interviewed each Extension professional three times over the course of the intervention: (1) baseline–within 4 weeks of session 1 of the program, (2) midpoint–after completing the first 6 months of the program (minimum of 16 weekly sessions), and (3) post intervention–within 4 weeks of completing the second 6 months of the program (minimum of 6 monthly sessions). Thus, interviews totaled 36 across the entire intervention with 12 per timepoint (baseline, midpoint, and post-intervention). Semi-structured interview guides were adapted from Damschroder et al. (2015), informed by all five CFIR domains, and included open-ended and select scaled (1–5) questions to evaluate Extension professionals’ experiences implementing the DPP in Georgia Extension over time. Interviews were audio recorded via Zoom Web Conferencing (Zoom Video Communications, 2021) and transcribed via a third-party transcriptionist (Rev.com, 2021).

### Data Analysis

Descriptive statistics were calculated for quantitative RE-AIM components. IBM SPSS version 27 was used for all quantitative data analysis (IBM, 2020).

Qualitative interview coding was primarily deductive, guided by the CFIR constructs, but allowed for inductive coding when the data did not fit the CFIR constructs. ATLAS.ti version 9 was used as a tool for qualitative coding and analyses (ATLAS.ti, 2019). Transcripts were reviewed by five analysts in pairs using a consensual qualitative approach (Damschroder et al., 2015, 2017a, b; Swindle et al., 2019). One researcher served as one of the two coders on all transcripts to provide consistency. Analyst pairs coded each transcript independently then met to review all codes, discuss, and reach consensus on any discrepancies.

Each construct within each transcript was then rated for its influence on implementation, using similar methods to (Damschroder & Lowery, 2013). Constructs were assigned both a valence (+ or –) and a magnitude (1 or 2) to indicate the direction and strength of influence on implementation, respectively. Ratings of “0” indicated a neutral influence on implementation, “X” a mixed influence, and “*” a slight influence in the direction specified (e.g., 1+*). For constructs missing within a transcript, “M” was assigned.

Following visual inspection of the data, constructs were identified as having “strong” influences on implementation if ratings were consistently positive (+ , facilitator) or negative (–, barrier) AND at least 25% (n = 3) of transcripts had a +2 or –2 rating. Constructs were considered to have “weak” influences if ratings were consistently positive (+) or negative (–) OR at least 25% (*n* = 3) of transcripts had a +2 or -2 rating for the construct. Magnitude and valence of all CFIR constructs and identification of those manifesting as “strong” or “weak” influencers of implementation are included in Supplemental File 1.

CFIR constructs that were determined to be “weak” and “strong” influencers of implementation were also assessed for how often the occurred (i.e., were coded together) with the RE-AIM domains to inform implementation barriers and facilitators to achieving each RE-AIM outcome (Supplemental File 2). Researchers coded each quote within each transcript with the RE-AIM domain(s) being described. CFIR constructs discussed more than 50% of the time in respect to a single RE-AIM domain are highlighted in the results.

## Results

A summary of the RE-AIM outcomes is presented in Table 1. Table 2 shows the RE-AIM domains, along with selected constructs that co-occurred at least 50% of the time with the RE-AIM domain. Table 2 also includes representative quotes to provide evidence of the construct as described by the Extension professionals (Tong et al., 2007). The following sections discuss the RE-AIM outcomes and select CFIR and researcher-developed constructs that manifested as strong implementation barriers (–) and facilitators (+). Note that the “barrier” and “facilitator” language throughout is used to describe Extension professionals’ discussion of the construct, not to describe a cause-effect relationship between the construct and outcomes. Constructs are designated in italics throughout.

**Table 2:** Select CFIR constructs that acted as barriers and facilitators to RE-AIM domains in the DPP

**RE-AIM domain**	**Construct**	**Barrier or facilitator**	**Representative quote**
**Reach**	*External change agents*	Facilitator (strong)	“Having the Wellness Coordinator…was extremely instrumental because…she's privy to everyone's blood tests, high-risk issues…So she's able to parlay that information into getting them in the pipeline” (Extension professional J)
	*Structural characteristics*	Mixed (weak)	“the hospital…I already had a working relationship with…I've already proven why Extension programs work, so just bringing in this list, ‘Hey, I have another resource.’” (Extension professional G)
**Effectiveness**	*Participant receptivity*	Facilitator (weak)	“seeing the bonding among the participants…that’s a huge accomplishment because some of my participants…really don’t get out, they’re older. That increases their quality of life…not just their health.” (Extension professional K) “the consistent attendance…even considering we went away from in person…the fact that people are still …actively participating in the groups would show a success.” (Extension professional B)
**Adoption**	*Patient needs and resources*	Facilitator (weak)	“I'm just doing it for the health of the people in my community.”(Extension professional B)
	*Intervention source*	Facilitator (strong)	“if it wasn't for…[state Extension Specialist], I wouldn't have known the program was out there so I would have never implemented the program…” (Extension professional E) “it being a CDC program. I mean, that comes with a lot of clout.” (Extension Professional A)
	*Tension for change*	Facilitator (weak)	“We've done a lot of programs on managing chronic disease, being on that preventative side is important.” (Extension professional H)
	*Evidence strength and quality*	Facilitator (strong)	“The evidence…the wide scope of it…how far reaching it was…[I]was like, ‘Oh my goodness, this is definitely something that all Extension offices should be offering because of the positive impact that it has made nationwide.’" (Extension professional D) “I'm about prevention rather than treatment, and I do believe that with healthy eating and regular physical activity you can prevent a lot of things'm (Extension professional F)
	*Compatibility*	Facilitator (strong)	“I think it aligns extremely well [with the Extension mission] because we’re taking research-based programming and this is one of the best research-based diabetes programs that there has been.” (Extension professional A)
	*Cost*	Barrier (weak)	“[The state Extension office covering the costs of training and printing] was huge because I certainly couldn’t have come up with that kind of money out of my [county] account.” (Extension professional J)
**Implementation**	*Networks and communications*	Facilitator (strong)	“it [the technical assistance calls] helps me to be more accountable and make sure that I am thorough in what it is I'm giving you, what you're asking for." (Extension professional D)
	*Learning climate*	Facilitator (weak)	“You guys are very approachable and…offer questions and concerns about the program…yourself. So, we don’t feel like we’re just barking at the wrong tree.” (Extension professional B)
	*Goals and feedback*	Facilitator (weak)	“there were clear goals set out there which makes it easy to know how and what needs to be done from my end to implementhe (Extension professional G)
	*Leadership engagement*	Facilitator (strong)	“I think it speaks very highly of [DPP coordinator] and [Specialist], because you are implementing the program…you have firsthand some of those same challenges that we have… It's not just the knowledge from research or the curriculum, you actually have firsthand knowledge.” (Extension professional K)
	*Formally appointed internal implementation leaders*	Facilitator (strong)	“The planning it took…this is where…having a Specialist…or having [a DPP coordinator] and the Specialist kind of helps.” (Extension professional G)
	*Opinion leaders*	Facilitator (strong)	
	*Champions*	Facilitator (strong)	
	*Access to knowledge and information*	Facilitator (weak)	“for something like this, I think anytime you can have [a registered dietitian] involved…you're definitely meeting a baseline need.” (Extension professional J)
	*Individual identification with organization*	Mixed (weak)	“being a CEC [County Extension Coordinator, administrative role], there's just always something. I don't know that I ever feel like, on a daily basis, I'm doing everything I could do to support my programing as effectively as I would like to.” “no matter what happens, I'm going to stay in touch with them and support them because I feel like I've made an obligation.” (Extension professional J)
	*Evidence strength and quality*	Facilitator (strong)	“I believe that it has been done and implemented for long enough that there is strong evidence that by following the program the way it's written, it does have value.” (Extension professional I)
	*Knowledge and beliefs about the intervention*	Mixed (strong)	“I find it fascinating that there's really no recipes……in general, I'm surprised…that we really don't talk about some of the nuts and bolts of nutrition a little bit more.” (Extension professional J) “the idea is that we spend at least one hour a week sitting with these folks and we've talked to them about fitness breaks…the lesson on fitness breaks incorporates getting up and moving. But none of the other lessons, I don't believe has a fitness break worked into it.” (Extension professional B)
	*Complexity*	Barrier (weak)	“starting to do the food logs and get feedback is going to be a little bit challenging.” (Extension professional A)
**Maintenance**	*Learning climate*	Facilitator (strong)	“I think that it’s something that…we’ve shown…successful…it…definitely falls in line with our mission…I think that it’s…definitely one that’s here to stay.” (Extension professional E)
	*Access to knowledge and information*	Facilitator (weak)	“there are things that we do need, so it’s not just give me the keys to the car.” (Extension professional B)

*CFIR* Consolidated Framework for Implementation Research, *RE-AIM* Reach, Effectiveness, Adoption, Implementation, and Maintenance

### Reach (R)

Of the 124 individuals that attended DPP informational sessions or otherwise expressed interest in participating in the DPP, five were ineligible due to T2DM diagnosis. The program reached 119 individuals (96%), and 71% (*n* = 88) were eligible and consented to participate in the research study (Table 1). Reach at each site ranged from 1 to 13 participants.

*External change agents* were community partners such as physicians, worksite wellness coordinators, and local news outlets (radio, newspaper, television) that, for most Extension professionals, promoted recruitment for the DPP. Extension professionals valued their community partners for promoting DPP reach.

*Structural Characteristics* of Extension acted as both barriers and facilitators of reach. Some Extension professionals discussed Extension’s established reputation as a health education provider and administrative structure, with county, district, and state leaders to provide programming support, including recruitment ideas, as DPP recruitment facilitators. However, others discussed poor visibility of Extension in some communities, particularly larger and more urban communities, as a recruitment barrier. Extension professionals noted that DPP reach would likely be limited to Georgia counties with Extension professionals who had a health and wellness assignment and that this ultimately impacted Extension’s reach and *Readiness for Implementation*.

*Time* was a primary barrier to reach. Extension professionals began recruiting in late October/early November for a January start date, with holidays presenting recruitment challenges. Most felt that at least 3 months and as many as 6 months may be needed to optimize reach.

### Effectiveness (E)

Mean weight loss (5.2 ± 5.0%) and physical activity (179 ± 122 min/week) exceeded the program goals. Nearly half (46.7%) and more than half (56.7%) of participants met the program weight and PA goals, respectively (Table 1).

*Participant receptivity* emerged as a subtheme of *patient needs and resources* to capture discussion around participants’ perception of DPP effectiveness and receptivity to/satisfaction with the DPP. Extension professionals discussed outcomes that they perceived to be additional indicators of program effectiveness, including self-reported reductions in hemoglobin A1c, and reduction or elimination of medications. Some Extension professionals spoke to the dynamic of their group, “bonding,” attendance, and retention as measures of DPP effectiveness.

Overall, *COVID-19* impeded implementation, including effectiveness. Further descriptions of the perceived influence of the COVID-19 pandemic on DPP participants’ health behaviors are reported elsewhere (Wilson et al., 2022).

### Adoption (A)

Of the 13 Extension professionals trained to deliver the DPP, 12 of 13 (92%) adopted the program and began implementation in winter 2020 as intended (Table 1). The non-adopting Extension professional relocated shortly after the training and began a cohort in fall 2020, though not included in the present study.

*Patient needs and resources* was the primary facilitator of DPP adoption, as every professional cited high T2DM rates and a need for T2DM prevention interventions in their communities. The DPP’s published *evidence strength and quality* for reducing T2DM risk and the “clout” that came with CDC being the *intervention source* encouraged them to adopt the program.

Extension professionals frequently discussed the *compatibility* of the DPP with Extension’s mission and values of evidence-based health promotion programs. Moreover, several professionals indicated the DPP addressed the *tension for change* within Extension toward more prevention-focused, evidence-based programs with measurable outcomes and funding potential, like the insurance reimbursement potential for DPP providers who obtain CDC recognition.

Multilevel *leadership engagement* was also instrumental in adoption. Extension professionals identified engagement from the state Extension Specialist in nutrition and health (*champion*), their district- and county-level extension leaders (*opinion leaders*), and the two Extension professionals that implemented the DPP prior to this project as facilitators of adoption.

*Cost* and *time* were primary barriers to adoption*.* While grant funding covered costs for these cohorts, Extension professionals spoke to *cost* as a potential barrier to adoption by others following this implementation project*.* the known *time* commitment to implement the year-long DPP and its *Complexity* emerged as barriers to adoption. However, many professionals stated a willingness to overcome these barriers due to the potential results.

### Implementation (I)

Extension professionals implemented the DPP according to CDC standards, as evidenced by the average number of sessions (26), their length, and the minimum number exceeding the 22 minimum session requirement (Table 1).

Several features of the DPP and implementation strategies used facilitated implementation. First, while Extension professionals felt that the program could be improved through activities like recipe and physical activity demonstrations, their knowledge of the DPP’s *evidence strength and quality* and the need to implement it as intended to achieve the intervention goals positively influenced fidelity in implementation. The clear program *goals and feedback* from leaders also facilitated implementation. Extension professionals felt that the program effectiveness goals (weight loss, PA, attendance) and implementation goals (meeting CDC recognition standards) were clearly defined and progress toward these goals was communicated back to the Extension professionals by leadership.

*Networks and communications* between Extension professionals and state Extension leaders and implementation strategies, such as technical assistance calls, that facilitated communication were discussed extensively as facilitators of DPP Implementation. Extension’s *Structural Characteristics* supported communication and implementation. Leaders engaged through regular emails, calls, and texts with Extension professionals to answer questions, solve problems, and provide other support as necessary. Extension professionals also spoke to the value of *access to knowledge and information* in the form of leaders with nutrition expertise (Extension Specialist in nutrition and health is a registered dietitian nutritionist) and with first-hand experience delivering the DPP. One of the two Extension professionals who had implemented the DPP prior to this implementation project spoke to the value of this support compared to when they were implementing the DPP on their own.

The complexity of the intervention and time and resources to implement were the primary barriers to implementation. Four Extension professionals served as County Extension Coordinators, which is an administrative role. These additional administrative responsibilities limited *time* and *available resources* they had to devote to DPP implementation, especially during the COVID-19 pandemic. Extension professionals noted the *complexity* of the DPP made implementation difficult, particularly the year-long duration, providing feedback on participants’ food records at each session, and completing make-up sessions for participants who missed sessions.

Extension professionals generally felt that the COVID-19 pandemic negatively influenced implementation of the DPP. Detailed descriptions of the influence of the COVID-19 pandemic on DPP implementation are reported elsewhere (Wilson et al., 2022).

### Maintenance (M)

At the conclusion of the present study (April 2021), five Extension professionals had begun six new DPP cohorts (virtual), one Extension professional had started one new in-person DPP cohort, and two Extension professionals planned to start new in-person cohorts in Fall 2021 (Table 1). Both programs were implemented as planned.

Extension professionals spoke to the *evidence strength and quality* of the DPP and its *compatibility* with Extension’s mission as facilitators of maintenance of the DPP in Extension. Some spoke to the potential for Medicare reimbursement giving the DPP a higher *relative advantage* compared to existing Extension health behavior change programs. Lastly, Extension professionals spoke to the value of implementing an evidence-based program like the DPP for demonstrating local impact for their promotion process (*organizational incentives and rewards*).

Extension professionals spoke to the need for continued *leadership engagement* in the form of involvement and support of leaders as the DPP is maintained in Extension. In particular, they discussed the value of the regular technical assistance calls for maintaining implementation.

Extension professionals also spoke to the value of communicating with other Extension professionals implementing the program (*agent networks*) through these calls and other avenues for DPP maintenance. Several suggested a mentorship program for Extension professionals new to implementing the DPP as essential to further dissemination and maintenance in Georgia Extension.

## Implications for Public Health

The present study provides important insights into implementation barriers, facilitators, and outcomes of the DPP in the context of a U.S. state Extension organization. RE-AIM outcomes were comparable to other DPP implementations (e.g., Damschroder et al., 2017a) and several constructs described as influential to implementation were identified using the CFIR framework.

### RE-AIM Outcomes of DPP Implementation in Georgia Extension

Reach of the DPP was positive (96%) compared to intended reach. Adoption was similar to Damschroder et al. (2017a) evaluation in a clinical setting, with a majority (92%) of trained individuals adopting the program. Basic assessment of implementation fidelity indicated that the program was implemented as intended. Lastly, maintenance in Georgia Extension is promising, with 8 of the 12 (67%) Extension professionals beginning or planning for another DPP cohort.

Barriers to and facilitators of reach identified through this trial can be utilized in future implementation to improve reach of the DPP in Georgia Extension. Effectiveness, adoption, implementation, and maintenance were respectable. Effectiveness measured by average weight loss and physical activity exceeded program goals and were similar to results of other research (Damschroder et al., 2017a, b; Ely et al., 2017; Gorczyca et al., 2022). All Extension professionals trained to implement the DPP eventually adopted the program, and a majority began another (and in some cases, multiple) DPP cohorts following conclusion of this study. These adoption and maintenance outcomes are noteworthy, considering their significance for T2DM prevention efforts in Georgia. Lastly, maintaining implementation fidelity in this community context of Extension, even during the COVID-19 pandemic, is of value not only for achieving the effectiveness outcomes observed in the present study, but also for informing DPP implementation fidelity in other community contexts where DPP adaptations are commonplace.

### CFIR Barriers and Facilitators Influencing RE-AIM Outcomes

This implementation evaluation identified several barriers and facilitators of DPP implementation in the context of Extension, some of which may be applicable to other community settings. Discussed facilitators of DPP reach included community partners and the rapport of Extension in communities, while limited *time* for recruitment was a barrier. These findings highlight the importance of Extension professionals having an established community presence and network of partners to promote recruitment. These findings are consistent with other studies using the CFIR to evaluate other community-based programs, including our own (King et al., 2019), reporting that programs with the highest referral rates were implemented by those who had strong community partner relationships (Damschroder et al., 2017a; King et al., 2019). In the case of the DPP, community partners help increase awareness of the program and clinical providers (physicians and nurses) can directly refer eligible patients. DPP dissemination and implementation efforts should consider allowing at least 3–4 months, and up to 6 months, for recruitment depending on the degree of implementers’ network in the community and existing referral structures. When medical providers are referring to the program, this time may be reduced depending on the provider’s volume of eligible participants.

The discussed role of *goals and feedback* in effectiveness highlights the importance of DPP leaders providing consistent feedback on cohorts’ progress and potential areas for improvement. Notably, the present study utilized the Data Analysis of Participants System to track participant attendance, weight, and physical activity data (Association of Diabetes Care and Education Specialists, 2021). Extension professionals highlighted the value of this system coupled with leadership feedback for keeping their cohort progressing toward program goals.

The DPP’s source and associated evidence base in CDC facilitated adoption, a finding consistent with the findings of Damschroder et al. (2017a). DPP marketing efforts to potential participants, community partners, and even potential program implementers should emphasize the DPP as a CDC, evidence-based program to improve buy-in. Extension professionals’ perceived “fit” of the DPP with Extension’s mission and programming, along with the present study’s high adoption rate, supports the value of Extension as a delivery system to increase dissemination of the DPP. When asked about increasing adoption of the DPP in other counties throughout the state, Extension professionals felt that success stories from the implementation pilot, the support provided for implementation, and the value of the DPP’s evidence base for building community rapport and Extension professionals’ impact statements would be incentives for adoption by other counties. Still, Extension professionals noted that adoption would be limited to counties with Extension professionals. With the number of county Extension professionals decreasing, considerations on how to maintain the strong adoption observed in the present study and how to promote reach throughout the state should be made in light of these realities. Delivery during the COVID-19 pandemic highlighted the value of virtual delivery for accessing residents in counties without county-based Extension professionals. Virtual delivery should be explored in the future to overcome potential adoption and reach barriers.

Extension leadership contributed to the professionals’ knowledge of the DPP and decision to adopt the program. Compared to other settings in which the DPP might be implemented, the support infrastructure of Extension further positions it to be a strong delivery system (Franz & Fahey, 2012; Franz & Townson, 2008; Franz et al., 2010). Most Extension organizations have a nutrition and/or health Extension Specialist that provides access to expertise in DPP-related content areas (Harden et al., 2019), administrative oversight, and implementation support. Still, depending on the Extension structure, some Extension Specialists are assigned to several programs and may have limited time to support a single, complex program like the DPP. This barrier is not specific to Extension, as Damschroder et al. (2017a) cited similar challenges in the VA context. Extension professionals discussed the need for a permanent DPP coordinator to assist the Extension Specialist to overcome this challenge. CDC does suggest that programs have an assigned DPP coordinator. In small organizations, this may be particularly challenging; but in larger organizations like Extension, a staff member or graduate student can be assigned to this role, as in the case of our study.

### Implementation Strategies Utilized

Implementation strategies, including technical assistance calls, created a positive *learning climate* that Extension professionals felt facilitated implementation. These results echo those reported by Damschroder et al., who also used bi-weekly meetings to provide pertinent updates and information and problem-solve issues (Damschroder et al., 2017a). Extension professionals also spoke to the value of the additional day of training held after the lifestyle coach training. Damschroder et al. also found leadership involvement and support to be one of the most important facilitators of DPP implementation in the VA context (Damschroder et al., 2017a). For multisite DPP delivery systems, additional training on implementation protocols specific to that delivery system may be beneficial for optimizing outcomes. Continued support from leaders in the form of consistent communication and continuing education were all cited as important components of implementation that would be important for maintenance as well as expansion of the DPP into other counties. These consistencies noted between the present and Damschroder et al., (2015, 2017a) studies indicate that the implementation strategies utilized in both (technical assistance calls, leadership involvement, training) may promote implementation outcomes across multiple contexts.

## Limitations and Strengths

The present study is not without limitations. Notably, no control or comparison group was included to allow for either comparison of implementation outcomes with and without the utilization of implementation strategies, or comparison of barriers and facilitators presented by the context of Extension compared to another context, limiting conclusions that can be made from the presented results. Still, comparisons to the most comparable literature to date (Damschroder et al., 2017a) have been made throughout. Many of the implementation strategies employed in this study involved state-level leadership support and training for Extension professionals. Withholding support and training from Extension professionals is not acceptable in the setting of Extension, making comparison of outcomes with and without these implementation strategies not feasible. Future studies should consider testing different implementation strategies side by side (e.g., one-on-one technical assistance verses group-based technical assistance) and/or comparison of implementation barriers and facilitators within and outside Extension.

In addition, the research team involved in data collection and analysis was heavily involved in supporting program implementation, potentially introducing researcher bias. However, the familiarity of the researchers with the implementation process offered a more comprehensive understanding of the topics discussed in interviews. Furthermore, three of the five data analysts were not involved in supporting implementation. Additionally, no objective measure of fidelity was included in the present study. Lastly, the number of counties/Extension professionals included in the present study was limited, compared to the total possible sample size in the state of Georgia. The initial sample was limited to meet financial constraints and assess initial feasibility in the pilot implementation study. Counties and Extension professionals from every region of the state, as well as both rural and urban counties, were included in an effort to increase the generalizability of the results.

There are also several strengths. This study is unique in its contribution to the literature by using standard frameworks (CFIR and RE-AIM) to rigorously evaluate implementation of an evidence-based program in a community setting that is well positioned to be an established DPP provider: Extension. Integration of the CFIR with RE-AIM also increases the translational value of this study, as the barriers and facilitators of RE-AIM identified using the CFIR in this study provide a foundation on which implementation strategies can be built to potentially enhance RE-AIM outcomes of the DPP in Extension and potentially other community contexts.

## Conclusions

Although freely available, the Diabetes Prevention Program is a complex intervention with many considerations for enhancing dissemination and implementation to reduce the public health burden of T2DM. Using the CFIR and RE-AIM frameworks, this study demonstrated similar reach, effectiveness, adoption and maintenance in Extension to DPP implementation in clinical contexts, and revealed Extension-system specific facilitators of RE-AIM outcomes. The supportive leadership structure, with state-level Extension Specialists and local community health educators (Extension professionals), compatible mission, access to content and implementation expertise, and established communication channels were discussed as benefits of this organizational structure. The strong Implementation, Adoption, and Maintenance observed in this study support the value of Extension as an effective and sustainable delivery system for the DPP. Future research should use similar methods to explore implementation in Extension and other contexts across the U.S. to further test the promising implementation strategies utilized in this study that promote communication and access to information, resources, and support to promote uptake and implementation of the DPP in Extension and beyond.

### Supplementary Information

Below is the link to the electronic supplementary material.

Supplementary file1 (DOCX 58 KB)

Supplementary file2 (DOCX 40 KB)

## Data Availability

The datasets generated and analyzed during the current study are available from the corresponding author upon reasonable request.

## Abbreviations

CDC: Centers for Disease Control and Prevention
CFIR: Consolidated Framework for Implementation Research
DPP: Diabetes Prevention Program
PA: Physical activity
RE-AIM: Reach, Effectiveness, Adoption, Implementation, Maintenance
StaRI: Standards for Reporting Implementation Studies
T2DM: Type 2 diabetes mellitus
VA: Veterans Health Administration
