# Correction: Implementation of the Diabetes Prevention Program in Georgia Cooperative Extension According to RE-AIM and the Consolidated Framework for Implementation Research

**DOI:** 10.1007/s11121-024-01665-y

**Published:** 2024-03-15

**Authors:** Hannah K. Wilson, Caroline Wieler, Darci L. Bell, Ajit P. Bhattarai, Isaura M. Castillo-Hernandez, Ewan R. Williams, Ellen M. Evans, Alison C. Berg

**Affiliations:** 1 Department of Nutrition, Dietetics and Exercise Science, Concordia College, Moorhead, MN 56562 USA; 2 Department of Nutritional Sciences, University of Georgia, Athens, GA 30602 USA; 3 Department of Organizational Learning and Performance, Idaho State University, Pocatello, ID 83209 USA; 4 Human Movement Sciences Research Center, School of Physical Education and Sports, University of Costa Rica, San José, 11502 Costa Rica; 5 Department of Health Sciences and Research, Medical University of South Carolina, Charleston, SC 29425 USA; 6 Department of Kinesiology, University of Georgia, Athens, GA 30602 USA

**Correction to: Prevention Science** 10.1007/s11121-023-01518-0

The article “Implementation of the Diabetes Prevention Program in Georgia Cooperative Extension According to RE-AIM and the Consolidated Framework for Implementation Research”, written by Wilson, H.K., Wieler, C., Bell, D.L., Bhattarai, A.P., Castillo‑Hernandez, I.M., Williams, E.R., Evans, E. M., and Berg, A.C., was originally published electronically on the publisher’s internet portal on 17 March 2023 without open access. With the author(s)’ decision to opt for Open Choice the copyright of the article changed on 15 February 2024 to © The Author(s) 2023 and the article is forthwith distributed under a Creative Commons Attribution 4.0 International License, which permits use, sharing, adaptation, distribution and reproduction in any medium or format, as long as you give appropriate credit to the original author(s) and the source, provide a link to the Creative Commons licence, and indicate if changes were made. The images or other third party material in this article are included in the article’s Creative Commons licence, unless indicated otherwise in a credit line to the material. If material is not included in the article’s Creative Commons licence and your intended use is not permitted by statutory regulation or exceeds the permitted use, you will need to obtain permission directly from the copyright holder. To view a copy of this licence, visit http://creativecommons.org/licenses/by/4.0/.

The original article has been corrected.

