# Supplementary material for: Implementation of the Diabetes Prevention Program in Georgia Cooperative Extension According to RE-AIM and the Consolidated Framework for Implementation Research
Source: Prev Sci. 2023 Mar 17;25(Suppl 1):34–45. doi: 10.1007/s11121-023-01518-0 (PMC10021035; doi:10.1007/s11121-023-01518-0)
Supplement: Supplementary file 1 — Supplementary file1 (DOCX 58 KB) [file 11121_2023_1518_MOESM1_ESM.docx]

| **Supplemental File 1. Consolidated Framework for Implementation Research (CFIR) and supporting implementation construct ratings of Extension Diabetes Prevention Program implementer interviews** | | | | | | | | | | | | | | |
| --- | --- | --- | --- | --- | --- | --- | --- | --- | --- | --- | --- | --- | --- | --- |
|  | **Interview Timepoint*** | **A** | **B** | **C** | **D** | **E** | **F** | **G** | **H** | **I** | **J** | **K** | **L** | **Overall Strength^** |
| **Intervention Characteristics** |  |  |  |  |  |  |  |  |  |  |  |  |  |  |
| Intervention Source | B | 2+ | 1+ | 2+ | 2+ | 1+ | 1+ | 2+ | 2+ | 2+ | M | 0 | M | Strong |
|  | M | 2+ | 1+ | M | M | 1+ | M | M | 0 | M | M | M | M |  |
|  | P | 2+ | 1+ | M | M | 1+ | M | M | M | M | M | M | M |  |
| Evidence Strength & Quality | B | 2+ | 1+ | 1+ | 1+ | 1+ | 2+ | 1+ | 1+ | 2+ | 1+ | 2+ | 1+ | Strong |
|  | M | 2+ | 1+ | 1+ | 1+ | 1+ | 2+ | M | X | 1+ | 1+ | 2+ | 1+ | Weak |
|  | P | 2+ | 1+ | 1+ | 1+ | 2+ | 1+ | 1+ | 1+ | 2+ | 1+ | M | 1+ | Weak |
| Relative Advantage | B | 2+ | 1+ | X | 1+ | 0 | 1+ | 1+ | 1+ | 0 | 0 | 1+ | 2+ |  |
|  | M | 0 | 1- | 1+ | 1+ | 1+ | X | 1+ | 1+ | 1- | X | 0 | X |  |
|  | P | M | 0 | 1+ | 2+ | 2+ | 2+ | X | 1+ | 2+ | 1+ | 1+ | 1+ | Weak |
| Adaptability | B | 1- | 1+ | 2- | X | 1+ | 1+ | 1+ | 1- | 1+ | X | 1+ | 1+ |  |
|  | M | 1- | 1- | 2- | 1+ | 1+* | 1- | 1+ | 1- | X | 1- | 1- | 1- |  |
|  | P | 1- | X | 2- | X | 1- | X | 1+ | 1+ | 1- | 1- | 1+ | 1- |  |
| Trialability | B | 1+ | 1+ | 1+ | 0 | 1+ | 1+ | 1+ | 1+ | 0 | 1+ | 0 | X |  |
|  | M | M | M | 1+ | M | M | M | 1+ | 1+ | 1+ | 1+ | M | M |  |
|  | P | M | M | 0 | M | M | M | 1+ | M | M | M | M | 0 |  |
| Complexity | B | 1- | M | 1- | M | 2- | 1- | M | X | 2- | 1- | 1- | 1+ |  |
|  | M | 1- | 1+ | 2- | 1- | X | 1-* | 1+ | 1- | 2- | 1- | 1-* | 1- |  |
|  | P | 1- | 1- | 2- | 1- | X | X | 1- | 1- | 1- | X | 1- | 1- | Weak |
| Design Quality & Packaging | B | 1- | X | 2- | 1- | X | 1+ | 2+* | 1- | X | 1- | 1- | 1+ |  |
|  | M | 1+* | 1- | 2- | X | 1+ | 1+ | X | 1- | X | 1- | 1- | X |  |
|  | P | X | 1- | 1- | 1+ | X | 1- | 1+ | 1- | 1- | 1- | 0 | 1- |  |
| Cost | B | 1- | M | 1- | 0 | 1- | 0 | 1- | 1- | 1- | 1- | 1- | 1- | Weak |
|  | M | 2+ | 1+ | 1+ | M | M | 1+ | 2+ | M | 1+ | 1+ | M | M | Weak |
|  | P | M | M | 1+ | 1- | M | M | M | M | 1- | 0 | M | M |  |
| **Outer Setting** |  |  |  |  |  |  |  |  |  |  |  |  |  |  |
| Patient Needs & Resources | B | 1+ | 2+ | 2+ | 1+ | 2+ | 2+ | 2+ | 1- | 1+ | 1+ | 1+ | 1+ | Weak |
|  | M | 1+ | X | 2- | 1- | 1+ | 1+ | X | 1- | 1+ | 1- | 1- | 1+ |  |
|  | P | X | 1- | 2- | X | X | 0 | 1-* | 1- | 1+ | 1+ | 1- | 0 |  |
| Cosmopolitanism | B | 0 | 1+ | 1+ | 1+ | 1+ | 2- | 1+ | X | 0 | 2+ | M | 1+ |  |
|  | M | 0 | 1+ | 1+ | 1+ | 0 | 1- | 1+ | 1-* | 0 | 1+ | M | 2+ |  |
|  | P | 1- | 0 | 0 | 1+ | 0 | 1- | 1+ | 0 | 0 | 1- | M | 1+ |  |
| Peer Pressure | B | 0 | 0 | 0 | 0 | 0 | 0 | 0 | 0 | 0 | 0 | 0 | 0 |  |
|  | M | M | M | M | M | M | M | M | M | M | M | M | M |  |
|  | P | M | M | M | M | M | M | M | 1- | M | M | M | 0 |  |
| External Policy & Incentives | B | 1+ | 1+ | X | 0 | 0 | X | 0 | X | 1+ | 0 | 1+ | 1+ |  |
|  | M | 1+ | 1+ | 2- | M | M | 1+ | M | X | M | X | M | M |  |
|  | P | M | M | 2- | M | 0 | M | M | M | 1- | 0 | X | 1- |  |
| **Inner Setting** |  |  |  |  |  |  |  |  |  |  |  |  |  |  |
| Structural Characteristics | B | 1+ | 1+ | 1+ | 1+ | 1+ | 1+ | 1+ | 1+ | 1+ | 2+ | 1+ | 0 | Weak |
|  | M | 2- | M | 1- | 1+ | 0 | 2+ | 2+ | 1+ | M | 1+ | 0 | 1+ | Weak |
|  | P | M | M | 1- | 1+ | M | 1+ | 2+ | M | 1+ | X | 1+ | M |  |
| Networks & Communications | B | 1+ | 1+ | 1+ | 2+ | 1+ | 2+ | 2+ | 1+ | 1+ | 2+ | 1+ | 1+ | Strong |
|  | M | 1+ | 1+ | 1+ | 2+ | 1+ | 1+ | 2+ | 1+ | 1+ | 1+ | 1+ | 1+ | Weak |
|  | P | 1+ | 1+ | 1+ | 1+ | 1+ | 1+ | 2+ | 1+ | 1+ | 1+ | 1+ | 1+ | Weak |
| Culture | B | 1- | M | 1- | 1+ | 1- | X | M | 0 | 1+ | X | 1- | 0 |  |
|  | M | 1- | 1+ | 1- | 1+ | 1+ | 2+ | X | 1+ | 1+ | 1+ | 1+ | X |  |
|  | P | 1- | 1+ | 1+ | 1+ | 1+ | 0 | 1+ | 1+ | 1+ | X | 1+ | 1+ |  |
| Implementation Climate | B | 1+ | 1+ | 2+ | 2+ | M | 1+ | 2+ | 1+ | 1+ | 1+ | 1+ | M | Strong |
|  | M | 1+ | M | M | 1+ | 2+ | 1+ | 1+ | 2+ | M | 0 | 1+ | 1- |  |
|  | P | 1+ | 1+ | 2+ | M | M | 1+ | M | M | 1+ | 1+ | 1+ | M | Weak |
| Tension for Change | B | 2+ | 1+ | 1+ | M | 1+ | 2+ | M | 1- | 2+ | 2+ | 2+ | M | Weak |
|  | M | 2+ | M | M | M | M | 1+ | M | M | M | M | M | M |  |
|  | P | M | M | M | M | M | M | M | M | M | 0 | M | M |  |
| Compatibility | B | 1+ | 2+ | 1+ | 2+ | 2+ | 1+ | 2+ | 1+ | 1+ | 2+ | 1+ | 2+ | Strong |
|  | M | 2+ | X | 1+ | X | 1+* | 1+ | 1+ | 1+ | 2+ | 1+ | 1+ | M |  |
|  | P | 1+ | 1+ | M | M | 1+ | M | 1+ | 1+ | 1+ | 2+ | 1+ | 1+ | Weak |
| Relative Priority | B | 0 | 1+ | 1+ | M | 2+ | 1+ | 1+ | M | 0 | X | 2+ | 1+ |  |
|  | M | 1+ | 1- | 1+ | 0 | 0 | 1+ | X | 1- | 1- | X | 0 | 1+ |  |
|  | P | M | X | M | M | M | M | M | 1+ | 1+ | M | 1+ | M |  |
| Organizational Incentives  & Rewards | B | 0 | 0 | 1+ | 0 | 1+ | 2+ | 1+ | 0 | 0 | 0 | 0 | 0 | Weak |
|  | M | 1+ | 1+ | 1+ | 1+ | 1+ | 1+ | 1+ | 1+ | 1+ | 1+ | 1+ | 1+ | Weak |
|  | P | 1+ | 2+ | 1+ | 0 | 1+ | 1+ | 1+ | 1+ | 1+ | 2+ | 2+ | 1+ | Strong |
| Goals & Feedback | B | 1+ | 1+ | 1+ | 0 | 2+ | 1+ | 2+ | X | 1+ | 1+ | 2+ | 0 | Weak |
|  | M | 1+ | 1+ | 1+ | 1+ | 1+ | 1+ | 1+ | 1+ | 1+ | 1+ | 1+ | 1+ | Weak |
|  | P | 1+ | 1+ | 1+ | 1+ | 1+ | 1+ | 1+ | 1+ | 1+ | 1+ | 2+ | 1+ | Weak |
| Learning Climate | B | 1+ | M | M | M | 1+ | 1+ | M | M | 1+ | 1+ | M | M |  |
|  | M | 1+ | 1+ | 1+ | 1+ | 1+ | 2+ | 1+ | 1+ | 1+ | 2+ | 1+ | 1+ | Weak |
|  | P | 1+ | 0 | 1+ | 2+ | 1+ | 2+ | 1+ | 1+ | 2+ | 2+ | 1+ | M | Strong |
| Readiness for Implementation | B | 2+ | M | 2+ | M | M | M | 1+ | M | M | M | 0 | M |  |
|  | M | X | 2+ | M | 1+ | 1+ | 1+ | 2+ | M | 2+ | M | 1- | M | Weak |
|  | P | M | M | M | X | 1+ | 2+ | M | M | M | 1- | M | M |  |
| Leadership Engagement | B | 1+ | 1+ | 1+ | 1+ | 1+ | 2+ | 2+ | 1+ | 2+ | 1+ | 1+ | 1+ | Strong |
|  | M | 1+ | 1+ | 2+ | 1+ | 1+ | 2+ | 2+ | 1+ | 2+ | 1+ | 1+ | 1+ | Strong |
|  | P | 1+ | 1+ | 2+ | 1+ | 1+ | 2+ | 2+ | 1+ | 1+ | 2+ | 1+ | 1+ | Strong |
| Available Resources | B | 1+ | 1+ | 1+ | X | 1+ | 1+ | 1+ | 1+ | 1+ | 1+ | 1+ | 1+ |  |
|  | M | 1- | 1+ | 1+* | 1+ | 1+ | 1+ | 2+ | 1+ | 1+ | X | 1+ | 1+ |  |
|  | P | 1- | 1+ | 1+ | 1+ | 1+ | 1+ | 1+ | 1+ | 1+ | 1- | 1+ | 1+ |  |
| Access to Knowledge  & Information | B | 1+ | 1+ | 1- | X | 1+ | 1+ | 1+ | 1+ | 1+ | 1+ | 1+ | X |  |
|  | M | 1+ | 1+ | 2+ | 1+ | 1+ | 1+ | 2+ | 1+ | 1+ | 1+ | 1+ | 1+ | Weak |
|  | P | 1+ | 1+ | X | 1+ | 2+ | 2+ | 1+ | 2+ | 1+ | 1+ | 1+ | 0 | Weak |
| **Characteristics of Individuals** |  |  |  |  |  |  |  |  |  |  |  |  |  |  |
| Knowledge & Beliefs  about the Intervention | B | 2+ | 1+ | 1+ | 1+ | 1+ | 2+ | 1+ | 1+ | 1+ | 1+ | 1+ | 2+ | Strong |
|  | M | 1+ | 1+* | 1- | 1+ | 1+ | 1+ | 1+ | X | 2+ | 1- | 1+ | 1+ |  |
|  | P | 1- | 1+ | 1+ | 1+ | 1+ | 2+ | 1+ | 1+ | 1+ | 1+ | 1+ | 1+ |  |
| Self-efficacy | B | 1+ | 1+ | X | 1- | X | 1+ | 1+ | 2+ | 1+ | M | 2+ | 1- |  |
|  | M | X | 1+ | 1+ | 2+ | 1+ | 1+ | 2+ | M | 1+ | 1+ | 1+ | 1- |  |
|  | P | M | 1+ | M | 1- | 1+ | 2+ | X | 1+ | 1+ | M | 1+ | 2+ |  |
| Individual Stage of Change | B | M | M | M | M | M | M | M | M | M | M | M | M |  |
|  | M | M | M | 2+ | M | M | 2+ | 2+ | M | 0 | M | 2+ | M | Strong |
|  | P | 1+ | 1+ | 1+ | 2+ | 1+ | 1+ | 1+ | 1+ | 1+ | 1+ | 1+ | 1+ | Weak |
| Individual Identification  with Organization | B | 1+ | 1+ | 1+ | 1+ | 0 | 1+ | 1+ | 1+ | 1+ | 1+ | X | 0 |  |
|  | M | X | 1+ | 2+ | 2+ | 1+ | 1+ | 2+ | 1+ | 1+ | 1+ | 1+ | M | Weak |
|  | P | 0 | 0 | 0 | 1+ | 0 | 1+ | 0 | 1+ | 0 | 0 | 1- | 0 |  |
| Other Personal Attributes | B | 2+ | 1+ | 2+ | 2+ | 1+ | 1+ | 1+ | X | 1+ | 1+ | X | X | Weak |
|  | M | 1+ | 1+ | 2+ | 1+ | 1+ | 1+ | 1+ | 1+ | 1+ | 1+ | 1+ | 1+ | Weak |
|  | P | 1+ | 0 | 2+ | 1+ | 1+ | 1+ | 1+ | 1+ | X | 1- | 1+ | 1+ |  |
| **Process** |  |  |  |  |  |  |  |  |  |  |  |  |  |  |
| Planning | B | 1+ | 1+ | 1+ | 1+ | 1+ | 1+ | 2+ | 1+ | 1- | 1+ | 1+ | X |  |
|  | M | 1+ | 1+ | 1+ | M | 2+ | 1+ | 1+ | 1+ | X | X | 1+ | M |  |
|  | P | M | 0 | M | 0 | 0 | 1+ | M | M | M | M | 1+ | M |  |
| Engaging | B | 1- | 1+* | 1+ | X | X | X | 1+ | 1- | 0 | 1- | 1+ | X |  |
|  | M | X | 0 | 1- | 1- | 1- | 2- | 1+ | 0 | M | 0 | 1- | 1+ |  |
|  | P | 1+ | 0 | X | 1+ | 0 | 1- | 1- | 1- | 0 | 1+ | 0 | 1- |  |
| Opinion Leaders | B | M | M | M | M | 2+ | 2+ | 2+ | 2+ | 2+ | 2+ | 1+ | 2+ | Strong |
|  | M | 1+ | M | M | M | 1+ | 2+ | 2+ | 1+ | M | X | 1+ | 1+ | Weak |
|  | P | M | M | M | 1+ | M | 1+ | 1+ | 1+ | 1+ | 1+ | M | 1+ |  |
| Formally Appointed Internal Implementation Leaders | B | 0 | 1+ | 1+ | 1+ | 1+ | 2+ | 2+ | 2+ | M | 1+ | 0 | 1+ | Strong |
|  | M | 1+ | 1+ | 1+ | 1+ | 1+ | 2+ | 1+ | 1+ | 1+ | 1+ | 1+ | 1+ | Weak |
|  | P | X | M | 1+ | 2+ | 1+ | 2+ | 1+ | 2+ | M | 1+ | 1+ | M | Weak |
| Champions | B | 1+ | 1+ | 1+ | 1+ | 2+ | 2+ | 2+ | 1+ | 1+ | 1+ | 1+ | 1+ | Strong |
|  | M | 2+ | 1+ | 1+ | 1+ | 1+ | 2+ | 2+ | 1+ | 1+ | 2+ | 1+ | 1+ | Strong |
|  | P | 1+ | 1+ | 1+ | 1+ | 1+ | 1+ | 1+ | 1+ | 2+ | 1+ | 1+ | 1+ | Weak |
| External Change Agents | B | M | 1+ | 2+ | 1+ | 1+ | 1- | 1+ | 1+ | 1+ | 2+ | 0 | 2+ | Weak |
|  | M | 0 | 1+ | 2+ | 1+ | 0 | M | 1+ | 0 | 0 | 2+ | M | 1+ | Weak |
|  | P | 1- | M | 0 | 1+ | M | 1- | 1+ | M | M | 2- | M | 2+ |  |
| Intervention Participants | B | 0 | 0 | 1- | 0 | 1- | 1+ | 1+ | X | 1+ | 1+ | 0 | 1+ |  |
|  | M | 0 | 0 | 1- | 0 | 1- | 1+ | 1- | 1- | 1+ | 1- | X | 1+ |  |
|  | P | 2- | 1+ | 1+ | 1+* | 1+ | 1+ | 1+ | X | 1- | X | 1+ | X |  |
| Executing | B | X | 1+ | 1+ | 1+ | X | 1+* | 1+* | 1+* | X | 1-* | X | 1+ |  |
|  | M | 1+ | 1+ | 1+ | 1+ | 1+ | 1+ | 1+ | 1+ | 1+ | 1+ | 1+ | 1+ | Weak |
|  | P | 1+ | 1+ | 1+ | 1+ | 1+ | 1+ | 1+ | 1+ | 1+ | 1+ | X | 1+ | Weak |
| Reflecting & Evaluating | B | 1+ | 0 | 1+ | X | 1- | 2- | X | X | 1- | 1- | X | 1+ |  |
|  | M | 1+ | 1+ | 0 | 1+ | 1+ | 1- | M | 1+ | 0 | M | M | 1+ |  |
|  | P | M | M | 1+ | 0 | M | 1+ | 2+ | M | X | 0 | 1+ | 1+ |  |
| **Other** |  |  |  |  |  |  |  |  |  |  |  |  |  |  |
| Implementation Strategy | B | 1+ | 1+ | 1+ | 1+ | 1+ | 2+ | 1+ | 2+ | 1+ | 1+ | 1+ | 1+ | Weak |
|  | M | 1+ | 1+ | 2+ | 1+ | 1+ | 1+ | 2+ | 1+ | 1+ | 1+ | 1+ | 1+ | Weak |
|  | P | 2+ | 1+ | 1+ | M | 2+ | 2+ | 2+ | 2+ | 1+ | 1+ | 1+ | 1+ | Strong |
| Agent Networks | B | 1+ | 1+ | 1+ | 2+ | 1+ | 2+ | 1+ | 1+ | 1+ | 1+ | 1+ | 1+ | Weak |
|  | M | 1+ | 1+ | 2+ | 1+ | 1+ | 1+ | 2+ | 1+ | 1+ | 1+ | 0 | 1+ | Weak |
|  | P | 1+ | 1+ | 1+ | 1+ | 2+ | 1+ | 1+ | 1+ | M | X | 1+ | M | Weak |
| Experience | B | 0 | M | 2+ | M | 2- | M | M | 1+ | 1+ | 1+ | 0 | M |  |
|  | M | 1+ | 0 | 2+ | 0 | 1+ | 1+ | 0 | 0 | X | 1+ | 1+ | 1+ |  |
|  | P | M | M | X | 0 | M | 0 | M | 1+ | 1+ | 1+ | 1+ | 0 |  |
| Facilitating | B | 0 | 0 | M | 1- | 2- | 0 | M | 1+ | M | M | 1- | 1+ |  |
|  | M | M | M | M | 1- | X | M | 1- | 1- | M | M | X | 0 |  |
|  | P | 2- | M | M | 1- | M | M | M | M | M | 1+ | M | 1+ |  |
| Time | B | 0 | 0 | 2- | 2- | 1- | 1- | 1- | 2- | 1- | 1- | 1- | 2- | Strong |
|  | M | 1- | 1+ | 1- | X | X | 1+ | 1+ | 1- | 1- | 1- | 1- | 1- |  |
|  | P | 1- | X | 1- | 1- | 1- | 1- | 1- | X | 1- | 1- | 1- | 1- | Weak |
| Participant Receptivity | B | 2+ | M | 1+ | 1+ | 1+ | 1+ | 1+ | X | 0 | 1+ | 1+ | 2+ |  |
|  | M | 2+ | 1+ | 1+ | 1+ | 1+ | 1+ | 1+ | 1+ | 1+ | 1+ | 1+ | 1+ | Weak |
|  | P | X | 1+ | 1+ | 1+ | 1+ | 1+ | 1+ | 1+ | 1- | 1+ | X | 1+ |  |
| Participant Networks | B | M | M | M | M | M | M | M | M | M | M | M | M |  |
|  | M | M | M | M | M | M | M | M | 2+ | M | M | M | 0 |  |
|  | P | 2+ | 2+ | M | 2+ | M | M | M | M | M | M | 2+ | M |  |
| Zoom | B | M | M | M | M | M | M | M | M | M | M | M | M |  |
|  | M | M | M | M | M | M | M | M | M | M | M | M | M |  |
|  | P | 0 | 1+ | 2- | M | M | M | 1+ | M | 2- | 1+ | 2- | 1+ | Weak |
| COVID | B | M | M | M | M | M | 2- | M | M | M | M | M | M |  |
|  | M | X | 1- | 1- | 1- | 1- | 1- | 1- | 1- | 1- | 1- | 1- | 1- | Weak |
|  | P | 1- | 1- | 1- | 1- | 0 | X | 1- | 1- | 1- | 1- | 1- | 1- | Weak |
| *B = baseline, M = midpoint, P = post-intervention  ^Strong = ratings were consistently positive (+) or negative (-) AND at least 25% (n=3) of transcripts had a +2 or -2 rating for the construct  Weak = ratings were consistently positive (1+) or negative (1-) OR at least 25% (n=3) of transcripts had a +2 or -2 rating for the construct | | | | | | | | | | | | | | |

|  | 2- |  | 1- |  | 0/M |  | X |  | 1+ |  | 2+ |
| --- | --- | --- | --- | --- | --- | --- | --- | --- | --- | --- | --- |
