# Supplementary material for: Implementation of the Diabetes Prevention Program in Georgia Cooperative Extension According to RE-AIM and the Consolidated Framework for Implementation Research
Source: Prev Sci. 2023 Mar 17;25(Suppl 1):34–45. doi: 10.1007/s11121-023-01518-0 (PMC10021035; doi:10.1007/s11121-023-01518-0)
Supplement: Supplementary file 2 — Supplementary file2 (DOCX 40 KB) [file 11121_2023_1518_MOESM2_ESM.docx]

| **Supplemental File 2. Frequency with which Consolidated Framework for Implementation Research (CFIR) constructs were coded together with RE-AIM domains in Implementer Interviews** | | | | | | | | | | | | | |
| --- | --- | --- | --- | --- | --- | --- | --- | --- | --- | --- | --- | --- | --- |
|  | **Interview Timepoint*** | **Strength** | **Construct Frequency^** | **Reach** | | **Effectiveness** | | **Adoption** | | **Implementation** | | **Maintenance** | |
|  |  |  |  | **#** | **%** | **#** | **%** | **#** | **%** | **#** | **%** | **#** | **%** |
| **Intervention Characteristics** | |  |  |  |  |  |  |  |  |  |  |  |  |
| Intervention Source | B | Strong | 19 | 1 | 5% | 0 | 0% | 19 | 100% | 0 | 0% | 0 | 0% |
| Evidence Strength  & Quality | B | Strong | 44 | 0 | 0% | 0 | 0% | 42 | 95% | 3 | 7% | 0 | 0% |
|  | M | Weak | 34 | 1 | 3% | 20 | 59% | 4 | 12% | 9 | 26% | 1 | 3% |
|  | P | Weak | 24 | 0 | 0% | 18 | 75% | 1 | 4% | 2 | 8% | 3 | 13% |
| Relative Advantage | P | Weak | 17 | 0 | 0% | 12 | 71% | 0 | 0% | 3 | 18% | 2 | 12% |
| Complexity | P | Weak | 93 | 4 | 4% | 12 | 13% | 0 | 0% | 74 | 80% | 6 | 6% |
| Cost | B | Weak | 18 | 0 | 0% | 0 | 0% | 15 | 83% | 4 | 22% | 0 | 0% |
|  | M | Weak | 21 | 0 | 0% | 2 | 10% | 7 | 33% | 13 | 62% | 2 | 10% |
| **Outer Setting** |  |  |  |  |  |  |  |  |  |  |  |  |  |
| Patient Needs & Resources | B | Weak | 67 | 7 | 10% | 1 | 1% | 57 | 85% | 7 | 10% | 1 | 1% |
| **Inner Setting** |  |  |  |  |  |  |  |  |  |  |  |  |  |
| Structural Characteristics | B | Weak | 14 | 1 | 7% | 0 | 0% | 12 | 86% | 1 | 7% | 0 | 0% |
|  | M | Weak | 24 | 6 | 25% | 0 | 0% | 8 | 33% | 9 | 38% | 3 | 13% |
| Networks & Communication | B | Strong | 59 | 2 | 3% | 0 | 0% | 3 | 5% | 55 | 93% | 0 | 0% |
|  | M | Weak | 98 | 1 | 1% | 0 | 0% | 2 | 2% | 98 | 100% | 0 | 0% |
|  | P | Weak | 56 | 0 | 0% | 0 | 0% | 0 | 0% | 53 | 95% | 4 | 7% |
| Implementation Climate | B | Strong | 52 | 3 | 6% | 0 | 0% | 46 | 88% | 3 | 6% | 0 | 0% |
|  | P | Weak | 15 | 0 | 0% | 1 | 7% | 0 | 0% | 8 | 53% | 6 | 40% |
| Tension for Change | B | Weak | 14 | 0 | 0% | 0 | 0% | 14 | 100% | 0 | 0% | 0 | 0% |
| Compatibility | B | Strong | 51 | 1 | 2% | 0 | 0% | 48 | 94% | 3 | 6% | 0 | 0% |
|  | P | Weak | 16 | 0 | 0% | 3 | 19% | 0 | 0% | 4 | 25% | 9 | 56% |
| Organizational Incentives & Rewards | B | Weak | 15 | 0 | 0% | 0 | 0% | 15 | 100% | 0 | 0% | 0 | 0% |
|  | M | Weak | 30 | 0 | 0% | 0 | 0% | 19 | 63% | 5 | 17% | 8 | 27% |
|  | P | Strong | 15 | 0 | 0% | 2 | 13% | 0 | 0% | 5 | 33% | 9 | 60% |
| Goals & Feedback | B | Weak | 20 | 0 | 0% | 1 | 5% | 0 | 0% | 19 | 95% | 0 | 0% |
|  | M | Weak | 72 | 0 | 0% | 11 | 15% | 0 | 0% | 60 | 83% | 1 | 1% |
|  | P | Weak | 36 | 0 | 0% | 4 | 11% | 0 | 0% | 31 | 86% | 1 | 3% |
| Learning Climate | M | Weak | 57 | 1 | 2% | 0 | 0% | 1 | 2% | 55 | 96% | 1 | 2% |
|  | P | Strong | 35 | 0 | 0% | 0 | 0% | 0 | 0% | 28 | 80% | 7 | 20% |
| Readiness for Implementation | M | Weak | 20 | 1 | 5% | 1 | 5% | 3 | 15% | 13 | 65% | 2 | 10% |
| Leadership Engagement | B | Strong | 74 | 4 | 5% | 0 | 0% | 35 | 47% | 38 | 51% | 0 | 0% |
|  | M | Strong | 135 | 3 | 2% | 0 | 0% | 7 | 5% | 124 | 92% | 2 | 1% |
|  | P | Strong | 75 | 0 | 0% | 3 | 4% | 1 | 1% | 64 | 85% | 10 | 13% |
| Access to Knowledge & Information | M | Weak | 116 | 1 | 1% | 0 | 0% | 7 | 6% | 108 | 93% | 2 | 2% |
|  | P | Weak | 52 | 0 | 0% | 4 | 8% | 1 | 2% | 41 | 79% | 7 | 13% |
| **Characteristics of Individuals** | | |  |  |  |  |  |  |  |  |  |  |  |
| Knowledge & Beliefs about the Intervention | B | Strong | 96 | 3 | 3% | 8 | 8% | 62 | 65% | 25 | 26% | 1 | 1% |
| Individual Stage of Change | M | Weak | 13 | 1 | 8% | 0 | 0% | 0 | 0% | 6 | 46% | 7 | 54% |
|  | P | Weak | 36 | 2 | 6% | 1 | 3% | 0 | 0% | 2 | 6% | 32 | 89% |
| Individual Identification with Organization | M | Weak | 39 | 1 | 3% | 1 | 3% | 7 | 18% | 24 | 62% | 6 | 15% |
| Other Personal Attributes | B | Weak | 133 | 18 | 14% | 6 | 5% | 61 | 46% | 55 | 41% | 0 | 0% |
|  | M | Weak | 171 | 6 | 4% | 10 | 6% | 17 | 10% | 141 | 82% | 7 | 4% |
| **Process** |  |  |  |  |  |  |  |  |  |  |  |  |  |
| Opinion Leaders | B | Strong | 18 | 3 | 17% | 0 | 0% | 11 | 61% | 5 | 28% | 0 | 0% |
|  | M | Weak | 19 | 0 | 0% | 0 | 0% | 5 | 26% | 14 | 74% | 0 | 0% |
| Formally Appointed Internal Implementation Leaders | B | Strong | 18 | 2 | 11% | 0 | 0% | 8 | 44% | 9 | 50% | 0 | 0% |
|  | M | Weak | 37 | 1 | 3% | 0 | 0% | 1 | 3% | 36 | 97% | 0 | 0% |
|  | P | Weak | 21 | 0 | 0% | 0 | 0% | 0 | 0% | 18 | 86% | 3 | 14% |
| Champions | B | Strong | 38 | 1 | 3% | 0 | 0% | 31 | 82% | 9 | 24% | 0 | 0% |
|  | M | Strong | 74 | 3 | 4% | 0 | 0% | 2 | 3% | 68 | 92% | 2 | 3% |
|  | P | Weak | 35 | 0 | 0% | 0 | 0% | 1 | 3% | 32 | 91% | 3 | 9% |
| External Change Agents | B | Weak | 47 | 43 | 91% | 0 | 0% | 2 | 4% | 5 | 11% | 0 | 0% |
|  | M | Weak | 21 | 13 | 62% | 0 | 0% | 0 | 0% | 7 | 33% | 2 | 10% |
| Executing | M | Weak | 327 | 12 | 4% | 64 | 20% | 1 | 0% | 263 | 80% | 1 | 0% |
|  | P | Weak | 210 | 1 | 0% | 62 | 30% | 0 | 0% | 147 | 70% | 9 | 4% |
| **Other** |  |  |  |  |  |  |  |  |  |  |  |  |  |
| Implementation Strategy | B | Weak | 46 | 3 | 7% | 0 | 0% | 0 | 0% | 43 | 93% | 0 | 0% |
|  | M | Weak | 60 | 1 | 2% | 0 | 0% | 0 | 0% | 60 | 100% | 0 | 0% |
|  | P | Strong | 29 | 0 | 0% | 0 | 0% | 0 | 0% | 26 | 90% | 4 | 14% |
| Agent Networks | B | Weak | 38 | 3 | 8% | 0 | 0% | 0 | 0% | 36 | 95% | 0 | 0% |
|  | M | Weak | 43 | 2 | 5% | 1 | 2% | 2 | 5% | 41 | 95% | 0 | 0% |
|  | P | Weak | 22 | 0 | 0% | 0 | 0% | 0 | 0% | 18 | 82% | 4 | 18% |
| Participant Receptivity | M | Weak | 148 | 10 | 7% | 57 | 39% | 2 | 1% | 78 | 53% | 7 | 5% |
| Time | B | Strong | 21 | 6 | 29% | 0 | 0% | 4 | 19% | 14 | 67% | 0 | 0% |
|  | P | Weak | 49 | 2 | 4% | 3 | 6% | 0 | 0% | 42 | 86% | 3 | 6% |
| Zoom | P | Weak | 21 | 1 | 5% | 2 | 10% | 0 | 0% | 16 | 76% | 3 | 14% |
| COVID | M | Weak | 160 | 8 | 5% | 28 | 18% | 2 | 1% | 123 | 77% | 3 | 2% |
|  | P | Weak | 107 | 0 | 0% | 16 | 15% | 0 | 0% | 86 | 80% | 7 | 7% |
| *B = baseline, M = midpoint, P = post-intervention  ^Total number of times the construct appeared across all interviews during that time point  Note: some CFIR constructs were coded with multiple RE-AIM domains and, thus, the sum of co-occurrence across a row may be greater than 100% | | | | | | | | | | | | | |

|  | CFIR construct and RE-AIM outcome coded together >50% of occurrences |  | CFIR construct and RE-AIM outcome coded together: 10 – 50% of occurrences |
| --- | --- | --- | --- |
